# Role of mitophagy in the neurodegenerative diseases and its pharmacological advances: A review

**DOI:** 10.3389/fnmol.2022.1014251

**Published:** 2022-10-04

**Authors:** Qixia Wang, Haoyuan Xue, Yundi Yue, Shiqi Hao, Shu-Hong Huang, Zhaoqiang Zhang

**Affiliations:** School of Clinical and Basic Medical Sciences, Shandong Provincial Hospital Affiliated to Shandong First Medical University, Jinan, China

**Keywords:** mitophagy, neurodegenerative diseases, Alzheimer's disease, Parkinson's disease, Huntington's disease, amyotrophic lateral sclerosis

## Abstract

Neurodegenerative diseases are a class of incurable and debilitating diseases characterized by progressive degeneration and death of cells in the central nervous system. They have multiple underlying mechanisms; however, they all share common degenerative features, such as mitochondrial dysfunction. According to recent studies, neurodegenerative diseases are associated with the accumulation of dysfunctional mitochondria. Selective autophagy of mitochondria, called mitophagy, can specifically degrade excess or dysfunctional mitochondria within cells. In this review, we highlight recent findings on the role of mitophagy in neurodegenerative disorders. Multiple studies were collected, including those related to the importance of mitochondria, the mechanism of mitophagy in protecting mitochondrial health, and canonical and non-canonical pathways in mitophagy. This review elucidated the important function of mitophagy in neurodegenerative diseases, discussed the research progress of mitophagy in neurodegenerative diseases, and summarized the role of mitophagy-related proteins in neurological diseases. In addition, we also highlight pharmacological advances in neurodegeneration.

## Introduction

As energy factories of cells, mitochondria generate enough energy through oxidative phosphorylation to maintain normal cellular processes. When cells are damaged, the number, size, and structure of mitochondria change to varying degrees. Mitochondria always exhibit balanced fusion, fission, and mitophagy, which are crucial for maintaining the integrity and function of mitochondria. Of these, mitophagy is the main mechanism for mitochondrial quality control (Cai and Tammineni, 2016; Rossmann et al., 2021).

Mitophagy, a kind of selective autophagy that clears damaged or dysfunctional mitochondria, is regulated by mitochondrial biogenesis, mitochondrial dynamics, calcium imbalance, mitochondrial DNA (mtDNA) damage, oxidative stress, and reactive oxygen species (ROS) (Ashrafi and Schwarz, 2013; Hirota et al., 2015; Chen et al., 2020). When mitophagy dysfunction occurs, severely damaged mitochondria cannot be removed promptly. This results in continuous accumulation of damaged mitochondria, and may even cause neuronal defects, thereby inducing Alzheimer's disease (AD), Parkinson's disease (PD), Huntington's disease (HD), amyotrophic lateral sclerosis (ALS), and other neurodegenerative diseases (Palikaras and Tavernarakis, 2012; Fivenson et al., 2017; Kerr et al., 2017; Liu et al., 2019; Cen et al., 2021).

## Mitophagy

Under the action of external stimuli such as ROS, nutritional deficiency, and cell aging, the mitochondria in cells are depolarized and damaged. Damaged or dysfunctional mitochondria are selectively removed *via* autophagosomes and then catabolized by lysosomes to maintain intracellular homeostasis, this process is known as mitophagy (Palikaras et al., 2018; Onishi et al., 2021). Mitophagy is the main mechanism of mitochondrial quality control and is mainly activated in mammalian cells by two pathways: PTEN-induced putative kinase 1 (PINK1) and Parkin (E3 ubiquitin-protein ligase parkin)–mediated mitophagy, which is ubiquitin-dependent, and a receptor-mediated mitophagy (Iorio et al., 2021).

### PINK1/parkin pathway in mitophagy

**Figure 1:**
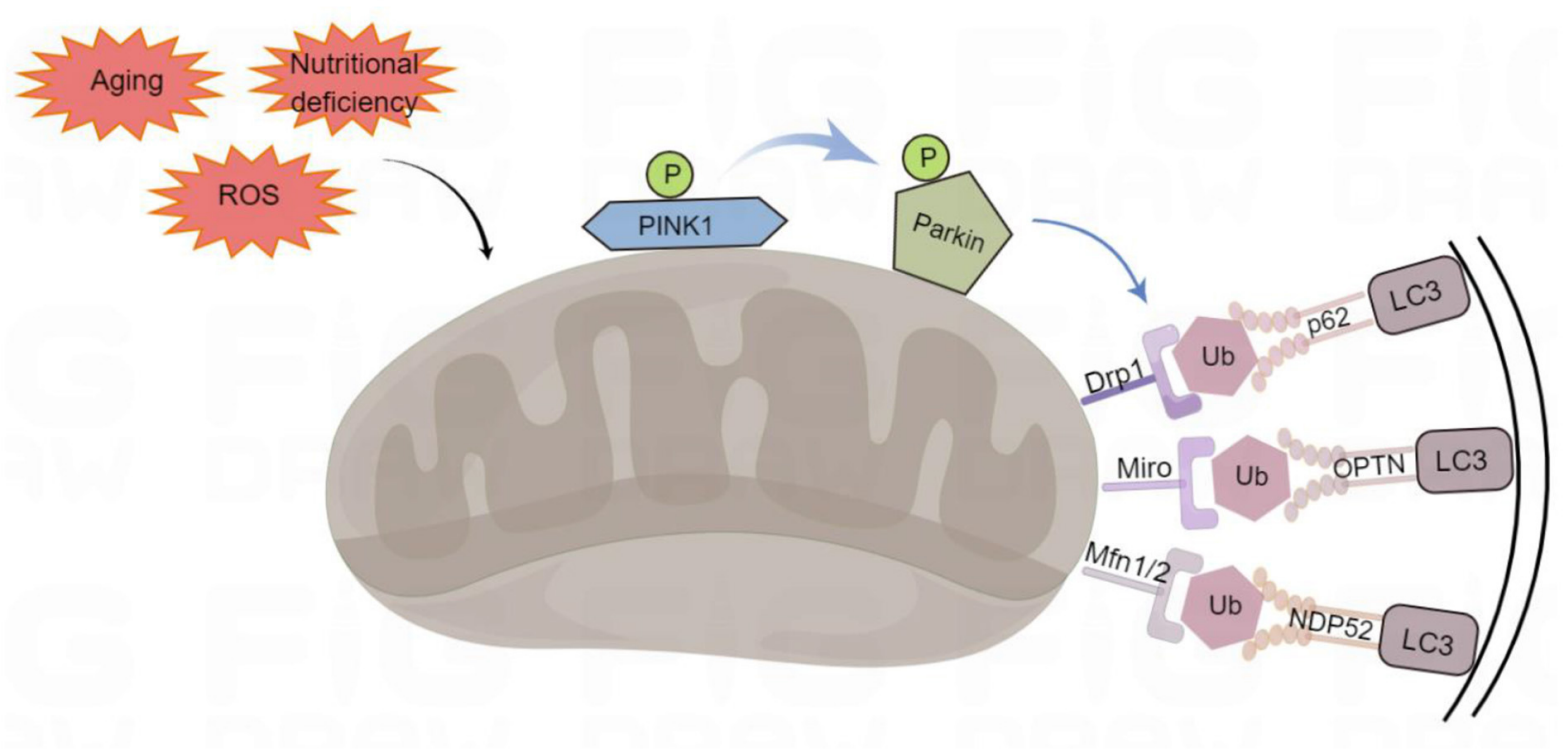
Ubiquitin-dependent mitophagy. PTEN-induced putative kinase 1 (PINK1) recognizes the damaged mitochondria, recruits Parkin to the damaged mitochondrial outer membrane, and phosphorylates it. Parkin can ubiquitinate proteins on the outer mitochondrial membrane, such as Mfn1/2 (mitofusins 1 and 2), Drp1 (dynamin-related protein 1), and Miro (mitochondrial Rho) to form polyubiquitin chains, which act as an “eat-me” signal for the autophagy mechanism to signal p62/SQSTM1 (sequestosome 1), optineurin (OPTN), and NDP52 (nuclear dot protein 52 kDa). They initiate mitophagy by LC3 (binding to light Chain 3) on autophagosomes through their LIR (LC3-interacting region) domains. By Figdraw (www.figdraw.com).

PINK1-Parkin-mediated mitophagy that advances mitochondrial degradation in a ubiquitin-dependent way is a canonical mitophagy pathway (Denisenko et al., 2021) (Figure 1). PINK1 is a mitochondrial serine/threonine kinase that serves as a molecular sensor of mitochondrial health, monitoring the health of mitochondria (Nguyen et al., 2016). In healthy mitochondria, newly synthesized PINK1 is continuously imported from the cytoplasm into the inner mitochondrial membrane, and its transmembrane domain is cleaved by the presenilin-associated rhomboid-like (PARL). After the processed PINK1 is released from the mitochondrial intermembrane space into the cytoplasm, it is rapidly degraded by the ubiquitin-proteasome system, which prevents PINK1 from accumulating on the mitochondrial outer membrane and reduces the level of PINK1 in healthy mitochondria, thus inhibiting mitophagy (Yamano and Youle, 2013). In damaged mitochondria, PINK1 accumulates on the mitochondrial outer membrane after depolarization of the membrane potential. The stable accumulation of several PINK1 molecules on the mitochondrial outer membrane leads to the translocation of Parkin to the damaged mitochondrial outer membrane. Parkin is an E3 ubiquitin ligase, localized in the cytoplasm, and PINK1 activates Parkin by phosphorylating its Ser65 residue leading to a conformational change (Bingol and Sheng, 2016). Moreover, PINK1 stimulates the phosphorylation of serine 65 on ubiquitin molecules, which promotes the recruitment and activation of Parkin and inhibits de-ubiquitination (Bingol and Sheng, 2016). Parkin acts as an amplifier of mitophagy. It ubiquitinates mitochondrial outer membrane proteins on dysfunctional mitochondria to form polyubiquitin chains, which are recognized by p62/SQSTM1 (sequestosome 1), nuclear dot protein 52 kDa (NDP52) and optineurin (OPTN) phosphorylated by PINK1 (Mizushima and Komatsu, 2011; Minowa-Nozawa et al., 2017). P62/SQSTM1, NDP52, and OPTN were formerly known as cargo adapters because they link autophagic cargo to LC3 during selective autophagy. They were more recently called autophagy receptors because they bind to the cargo and/or cause degradation. They recognize phosphorylated polyubiquitin chains on mitochondrial proteins and bind to the autophagosome marker microtubule-associated protein light chain 3 (LC3) through the LC3-interacting region (LIR) motif to initiate autophagosome formation, which associates with the lysosome fusion to initiate degradation of damaged mitochondria by autolysosomes (Lazarou et al., 2015).

P62/SQSTM1, containing ubiquitin-associated and LIR domains, is a selective cargo receptor for autophagic degradation of misfolded proteins (Liu et al., 2017). Initially, p62/SQSTM1 was considered to be the main mitophagy adaptor. However, later studies reported that p62/SQSTM1 did not play a decisive role and was dispensable for the occurrence of mitophagy (Narendra et al., 2010). In addition, the promoter region of p62/SQSTM1 contains antioxidant response elements (AREs). Therefore, the expression of p62/SQSTM1 can be upregulated by inducing AREs to induce mitophagy.

OPTN is highly expressed, whereas NDP52 is barely detectable in the brain. Interestingly, OPTN-mediated mitophagy appears to be spatially confined to neuronal cell bodies, with just a few mitophagy events observed in axons or dendrites (Evans and Holzbaur, 2020). OPTN can compensate for NDP52 by tank-binding kinase 1 (TBK1; a kinase involved in innate immunity that phosphorylates cargo adapters) during mitophagy. Previous research suggests that TBK1 improves the cargo adapter binding affinity to ubiquitin chains by phosphorylation and enhances mitophagy (Pickles et al., 2018).

### Mitophagy mediated by mitophagy receptors

**Figure 2:**
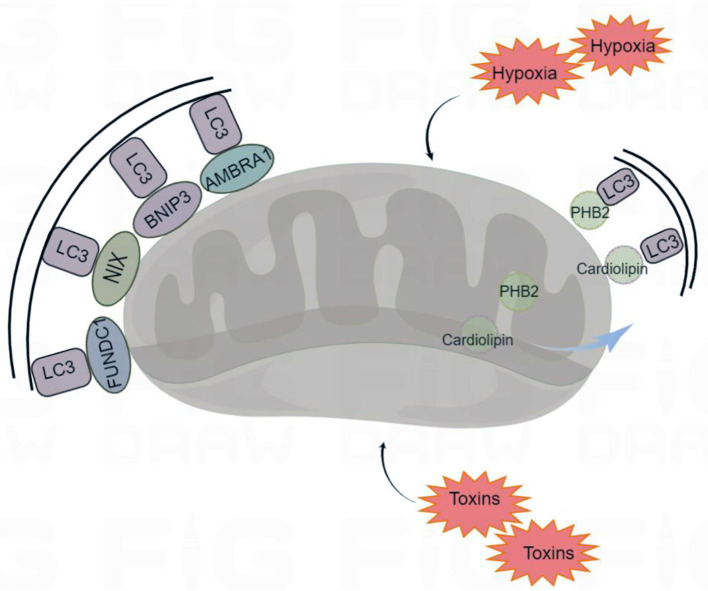
Receptor-mediated mitophagy. Under certain conditions (hypoxia and toxins) mitophagy receptors are activated. The mitophagy receptors containing LIR (LC3-interacting region) motifs, such as FUNDC1 (FUN14 Domain Containing 1), BNIP3 (BCL2 Interacting Protein 3), NIX (Nip3-like protein X), AMBRA1 (Activating Molecule in Beclin1-regulated Autophagy), PHB2 (Prohibitin 2), and cardiolipin, interact with the autophagosome light chain 3 (LC3), targeting the damaged mitochondria for mitophagy. By Figdraw (www.figdraw.com).

Mitophagy receptors usually refer to the mitochondrial proteins containing LIR motifs, which can interact with LC3. The most studied mitophagy receptors are the activating molecules in beclin1-regulated autophagy (AMBRA1), Bcl-2 and adenovirus E1B 19 kDa-interacting protein 3 (BNIP3), NIP3-like protein X (NIX), FUN14 domain containing 1 (FUNDC1), prohibitin 2 (PHB2), and cardiolipin (Zhang et al., 2008; Feng et al., 2013; Liu et al., 2014; Swerdlow and Wilkins, 2020) (Figure 2).

AMBRA1, NIX, FUNDC1, and BNIP3 are localized to the mitochondrial outer membrane. AMBRA1 can interact with Parkin to promote the occurrence of mitophagy. In the absence of Parkin and p62/SQSTM1, AMBRA1 can also act as a mitophagy receptor and bind to LC3 through its LIR motif to induce mitophagy (Strappazzon et al., 2015). NIX, which is involved in the clearance of mitochondria during mammalian erythrocyte maturation, plays an important role in mitophagy (Novak et al., 2010). Under hypoxia, the transcription of BNIP3 and NIX is activated by hypoxia-inducible factor 1-α (HIF1-α) and forkhead box class-O family member proteins (FoxO), and the activities of BNIP3 and NIX are regulated by phosphorylation. Phospholipids can increase their ability to bind to LC3 (Marinkovi and Novak, 2021). FUNDC1 is a new hypoxia induced mitophagy receptor. Its N-terminal LIR domain is exposed to the cytoplasm that can selectively respond to ischemia and hypoxia stimuli but not starvation stimuli. Phosphatase can change the phosphorylation state of the FUNDC1 site, which affects the binding affinity of the LIR motif to LC3, promoting or inhibiting mitophagy (Liu et al., 2012).

PHB2 and cardiolipin are localized to the inner mitochondrial membrane. When mitochondria are damaged, PHB2 and cardiolipin move to the outer mitochondrial membrane and interact with activated LC3, targeting the damaged mitochondria for mitophagy (Liu et al., 2019). Overall, mitophagy receptors are inactive at resting potentials, and their activity is elicited under various conditions. Moreover, various mitophagy receptors ensure specificity for various tissues and stimuli.

## The relationship between mitophagy and neurons

Neurons are terminally differentiated cells that can live through a lifetime. For this, they require energy in large amounts. Therefore, neurons are highly dependent on mitochondria. Besides, the strict control of mitochondrial size, number, and distribution is crucial for neurons (Millecamps and Julien, 2013; Schönfeld and Reiser, 2013; Stavoe and Holzbaur, 2019). Given the importance of mitochondria, it is necessary to understand the mechanism by which mitophagy protects neurons (Doxaki and Palikaras, 2020).

According to the physiological conditions of cells, mitophagy can be divided into programmed mitophagy, basal mitophagy, and stress-induced mitophagy. Activated mitophagy under stressors such as oxidative stress, starvation, and hypoxia reduces oxygen consumption and reactive oxygen species production in damaged mitochondria (Gaetano et al., 2021). The regulation of mitophagy varies by tissue, and in the context of stressors, mitophagy may be upregulated to increase mitochondrial function. Different stressors that induce autophagy can increase ROS production, such as starvation, heat shock, pathogen infection, and hypoxia. However, given the high reactivity of these stressors in biological systems, it is difficult to distinguish which are the key molecules that trigger mitophagy. Mitochondrial dysfunction increases oxidative stress and cellular damage in mammals, injured mitochondria can be eliminated through a PINK1-Parkin-dependent pathway or activation of mitophagy receptors, thereby reducing mitochondrial production of ROS. The architecture of neuronal cells characterizes the regulation of mitophagy in neurons, and damaged organelles are reversibly transported into the neuronal soma. Compared with neurons, astrocytes appear to be characterized by more pronounced changes in the mitophagy-regulated PINK1-parkin pathway in response to various stimuli, including inflammatory and metabolic stress (Sukhorukov et al., 2021).

The antioxidant responsive element (ARE) mediates transcriptional activation of protective genes, the transcription factor NF-E2-related factor 2 (Nrf2) maintains redox homeostasis by regulating the expression of ARE-dependent transcripts (Bahn and Jo, 2019). The Nrf2-ARE pathway serves as an indicator of oxidative stress in neurodegeneration, and the binding of Nrf2 to ARE protects cells from oxidative stress-induced cell death, which in the pathogenesis of multiple chronic neurodegenerative diseases The increase is associated with neuronal cell death (Raghunath et al., 2018). Neurons are the cells most vulnerable to excess reactive oxygen species because they have the highest energy demands and are more vulnerable than astrocytes (Barreto et al., 2012). Under conditions of aging and stress, neurons increase oxidative phosphorylation (OXPHOS) by enhancing glycolysis in astrocytes to meet the increased demand for lactate in neurons (Pellerin et al., 2007). Enhancing astrocyte function could represent a valuable neuroprotective strategy. In previous studies, selective overexpression of Nrf2 in astrocytes is an effective method to preserve neuronal viability in mice. The Nrf2-ARE pathway plays a neuroprotective role in glial cells, but the mechanism is also elusive and needs to be continued in neurodegenerative disease models such as AD, PD, HD, and ALS to investigate how this pathway mitigates neurodegeneration (Johnson et al., 2008).

Under physiological conditions, fusion is a way of changing mitochondrial morphology to enable the exchange of mtDNA, proteins, lipids, and metabolites, thereby avoiding the accumulation of dysfunctional mitochondria. Mitochondrial fission can release damaged mitochondria through mitophagy, which is effective in maintaining the quantity and quality of mitochondria (Youle and van der Bliek, 2012). Impaired mitochondrial dynamics (fusion and fission) may disrupt mitophagy, which increases oxidative stress and exacerbates neuronal degeneration (Knott et al., 2008). Mitochondria are particularly abundant at synapses, and impairment of their movement disrupts the synaptic transmission between neurons. Parkin ubiquitinates Rho GTPase (Miro), mitofusin1, and 2 (Mfn1/2), targeting them for degradation. Mfn1/2 degradation leads to blocked mitochondrial fusion, whereas Miro degradation prevents mitochondrial movement and isolates mitochondrial damage to specific regions for the occurrence of mitophagy. Therefore, accumulation of Parkin in mitochondria and degradation of Miro may serve as a protective mechanism for neurons (Sandoval et al., 2014).

The body normally clears dysfunctional mitochondria through mitophagy. The accumulation of injured mitochondria surveyed in neurodegenerative diseases, suggests that damaged mitochondria are not removed promptly and the processes of mitochondrial quality control are affected in these diseases (Mani et al., 2021). Studies have reported that impaired mitophagy is associated with various neurodegenerative diseases, such as AD, PD, HD, and ALS. The toxicity of pathogenic proteins in the mitophagy system may be a leading cause of neurodegenerative diseases, and the accumulation of pathogenic proteins [e.g., β-amyloid (Aβ), α-synuclein, and mutated huntingtin] often results from deficiencies in mitophagy (Chiang et al., 1989). The accelerated elimination of damaged mitochondria or misfolded proteins by enhancing mitophagy has been predicted as a novel approach to treating neurodegenerative diseases.

## Mitophagy and Alzheimer's disease

Alzheimer's disease (AD) is characterized by extracellular Aβ deposition and the formation of intraneuronal neurofibrillary tangles that ultimately lead to neuronal loss and cognitive deficits. (Ingelsson et al., 2004; Wang et al., 2020).

Mitochondria are the key organelles in AD pathogenesis. Their dysfunction can significantly contribute to early-stage AD progression. Impaired mitochondrial function can lead to energy deficiency, intracellular calcium imbalance, and oxidative stress, which exacerbates Aβ accumulation and tau hyperphosphorylation. This further contributes to cognitive decline and memory loss. Amyloid precursor protein (APP) is one vital player in AD, and Aβ is a cleavage product of the universally expressed APP. Prior work suggests that APP is involved in the pathology of AD (Manocha et al., 2016). Aβ, an important pathological protein found in the brains of patients with AD, was shown in mutant APP (mAPP) mice, suggesting that a lack of PINK1 promotes the accumulation of Aβ (Du et al., 2017). Tau is the number of the microtubule-associated protein (MAP) family and plays a significant role in promoting axonal transport, synaptogenesis, and neurite outgrowth. Furthermore, it is essential for maintaining cell structure and integrity. In AD, tau accumulates excessively in neurons, leading to abnormal tau phosphorylation and microtubule disassembly (Puri et al., 2009; Guha et al., 2020).

Mitochondrial quality control can be preserved through the balance of mitochondrial dynamics, which means that mitochondria are constantly fused and fissioned in the body to maintain cellular metabolic stability. In AD, the levels of fission proteins Fis1and Drp1 increase, whereas those of fusogenic proteins OPA1, Mfn1/2 decrease. This leads to increased mitochondrial fission and reduced fusion. This suggests that mitochondrial dynamics are severely impaired in AD (Zhu et al., 2013). The increase in Aβ increases nitric oxide levels in the brain, which in turn activate Drp1 and Fis1. This leads to excessive mitochondrial fragmentation and defective mitochondrial transport to synapses, which results in synaptic dysfunction. Further studies have reported that p-tau interacts with Drp1, enhances the enzymatic activity of the GTPase Drp1, and leads to excessive mitochondrial fragmentation and mitochondrial dysfunction in AD. The oligomers of Aβ and tau can be localized to mitochondria in the early stage of disease progression, altering mitochondrial morphology as observed in the model systems of several pathophysiological features (Manczak et al., 2006; Del Prete et al., 2017).

The most consequential damage caused to mitophagy in AD is the reduction in PINK1 and Parkin protein levels, which results in decreased mitophagy and accumulation of dysfunctional mitochondria (Oliver and Reddy, 2019). A previous study reported decreased levels of PINK1 protein and an increase in mitochondrial number in the hippocampal neurons in a mouse model of AD (Manczak et al., 2018). The overexpression of Parkin reduces Aβ plaques and amyloid-induced inflammation in the hippocampus and cortex in a mouse model of AD, which alleviates behavioral abnormalities (Hong et al., 2014). In both, early- and late-onset AD, defective mitophagy leads to synaptic dysfunction and cognitive deficits by triggering Aβ and p-tau accumulations. Studies on cell and animal models of AD and patients with AD support that impaired mitophagy increases Aβ and tau accumulations, thus aggravating AD synaptic defects and cognitive decline (Du et al., 2017; Kerr et al., 2017). Mice lacking PINK1 seem to develop Aβ plaques and mitochondrial abnormalities earlier, while PINK1 overexpression promotes the removal of damaged mitochondria and reduces synaptic defects and cognitive decline in mice (Du et al., 2017). Enhancing mitophagy can not only inhibit Aβ and tau protein aggregation but also reverse cognitive deficits in AD models. Thus, the regulation of mitophagy may be a new approach to treating AD.

## Mitophagy and Parkinson's disease

Parkinson's disease (PD) is characterized by gradually losing dopaminergic neurons in the substantia nigra pars compacta and selective loss of Lewy bodies. Although the exact pathogenesis of PD is still vague, substantial evidence shows that dysfunctional mitophagy, excess ROS, and mtDNA damage may work in PD development (Federico et al., 2012; Ryan et al., 2015).

The neuronal apoptosis in patients with PD can be affected by regulating mitophagy, which suggests that mitophagy may be closely related to PD. PINK1 and Parkin are the most widely studied PD-related proteins that can maintain mitochondrial function by participating in mitophagy, and their loss of function is the most widely known cause of autosomal recessive and early-onset PD (Valente et al., 2004). PINK1/Parkin mutations prevent Parkin recruitment to mitochondria and promote the number of unhealthy mitochondria. This may raise ROS and then promote PD lesions. This supports the earlier observations of the accumulation of damaged mitochondria in patients with PD.

Except for PINK1 and Parkin, most PD-related genes have a direct or indirect effect on mitochondrial dynamics and quality control. The mutations in α-synuclein and glucocerebrosidase (GBA) lead to mitochondrial dysfunction and increased or impaired mitophagy respectively. As an abundant neuronal protein in the brain, α-synuclein is related to the disruption of mitochondrial function and can maintain the normal function of synapses under normal conditions. Accumulated pathological α-synuclein binds preferentially to mitochondria, inhibits mitochondrial protein import, and causes depolarization of mitochondrial membrane and impairment of cellular respiration (Wang et al., 2019). DJ-1, encoded by the *PARK7* gene, is a mitochondria-localized redox sensor whose deletion impedes mitophagy and affects mitochondrial dynamics in mouse embryonic fibroblasts. Thus, it plays a key role in mitochondrial homeostasis (Gao et al., 2012). Mutations in DJ-1 cause mitochondrial dysfunction and oxidative stress, which are associated with autosomal recessive PD (Hague et al., 2003).

The pathogenic causes of PD are complex and still unknown. No effective strategy is available to fundamentally cure PD. Therefore, the enhancement of mitophagy to remove dysfunctional mitochondria serves as a potential strategy for the treatment of PD (Luo et al., 2015).

## Mitophagy and Huntington's disease

Huntington's disease (HD) is an autosomal dominant disorder characterized by progressive motor, cognitive, and psychiatric impairments (Ross and Tabrizi, 2011; Jimenez-Sanchez et al., 2017). HD is caused by the inheritance of Huntington gene mutation. The abnormal expansion of CAG trinucleotide repeats in the HTT gene is the genetic basis of HD and changes the conformation of HTT protein and produces neural toxicity, leading to neurodegenerative changes (Ross and Tabrizi, 2011; Podvin et al., 2019).

In neurons, huntingtin protein (HTT) performs essential functions in the control of intra-axonal organelle trafficking through the formation of complexes with huntingtin-associated protein 1 (HAP1), kinesin, and dynein (Wong and Holzbaur, 2014). Some evidence suggests that mutant huntingtin (mHTT) can interact with the mitochondrial outer membrane, which leads to mitochondrial abnormalities (Choo et al., 2004). MHTT is ubiquitous in the brain and peripheral tissues, and its ability to form insoluble aggregates and interact with other proteins results in transcriptional dysregulation, abnormal synaptic transmission, defects in cellular trafficking, and several cell dysfunctions (Franco-Iborra et al., 2021). In the presence of mHTT, impaired autophagosome trafficking increases the number of autophagosomes and fragmented mitochondria. MHTT interacts with the GTPase Drp1 and inhibits mitochondrial biogenesis, promoting synaptic degeneration in HD. Additionally, the enhanced mitochondrial biogenesis in the mouse models of HD reduced the HD phenotype (Chandra et al., 2016).

MHTT interacts abnormally with proteins in the mitophagy pathway, such as Unc-51-like autophagy-activating kinase 1(ULK1), beclin 1(BECN1), OPTN, NDP52, p62/SQSTM1, and Neighbor of Brca1(NBR1), resulting in reduced LC3 recruitment to damaged mitochondria, which in turn disrupts mitophagy. This implies that the rescue of mitophagy can prevent neuron loss, thereby slowing the disease onset (Franco-Iborra et al., 2021).

## Mitophagy and amyotrophic lateral sclerosis

As a fatal motor neuron disease, amyotrophic lateral sclerosis (ALS) is characterized by degenerative changes occurring in upper and lower motor neurons (Rowland and Shneider, 2001). The exact mechanism by which ALS occurs is unclear. However, ALS is known as a disorder of protein homeostasis. Mitophagy defects act a substantial role in the pathogenesis of ALS (Wong and Holzbaur, 2015).

The majority of ALS-related genes are involved in the regulation of mitophagy, such as superoxide dismutase 1 (*SOD1*), *OPTN*, TAR DNA-binding protein 43 (*TDP-43*), and *TBK1* (Rosen et al., 1993; Amin et al., 2020; Liu et al., 2021). Furthermore, misfolded or aggregated protein products of some ALS-causing genes (e.g., *SOD1*) that are not directly involved in mitophagy can interact abnormally with mitophagy proteins, thereby deregulating their activity (Zhang et al., 2007; Tak et al., 2020).

ALS-related mitochondrial dysfunction manifests itself in various forms, including defective oxidative phosphorylation, ROS production, impaired calcium buffering capacity, and defective mitochondrial dynamics. The accumulation of fragmented mitochondria is one of the common features of ALS, and it may indicate that mitophagy fails to remove damaged mitochondria (Smith et al., 2019). Some ALS-related mutations cause malfunctioning of proteins, such as OPTN, p62, or TBK1, and lead to inefficient mitophagy. For example, missense mutations in *TBK1* have various biophysical and biochemical effects on this molecule and are associated with ALS linked to frontotemporal dementia (Harding et al., 2021).

The accumulation of neurotoxic misfolded proteins and aggregates within motor neurons are the main pathological hallmarks of ALS. Mitophagy may decrease protein aggregates, and the induction of mitophagy may improve ALS pathology (Amin et al., 2020).

## Pharmacological advances

Mitochondrial damage is a hallmark of neurodegenerative disorders, such as PD and AD. Enhancing the levels of molecules that remove defective mitochondria may have significant therapeutic benefits (Mattson et al., 2008; Fang et al., 2017). Several mitophagy enhancers have been identified, such as NAD^+^ precursor, urolithin A (UA), actinin (AC), and spermidine. All of them can enhance mitophagy and increase mitochondrial response to oxidative stress, thus protecting human neurons and extending the lifespan of animal models (Gupta et al., 2013; Madeo et al., 2018).

### Mitophagy enhancers

As neurons consume large amounts of energy, they are sensitive to reduced NAD^+^ levels and reduced ATP production (Fang et al., 2016). NAD^+^ affects neuronal health and survival, whereas reduced NAD^+^ levels impair mitophagy, facilitate the backlog of misfolded proteins, and even lead to neuronal death (Zhou et al., 2015). The major NAD^+^ precursors include nicotinamide riboside, nicotinamide, and nicotinamide mononucleotide. NAD^+^ levels are reduced in the animal models of AD, and cellular NAD^+^ levels are increased by supplementing NAD^+^ precursors to alleviate Aβ and tau lesions while preventing cognitive impairment (Brown et al., 2014; Scheibye-Knudsen et al., 2014). UA, an ellagitannin-derived metabolite, induces mitophagy in neurons of *Caenorhabditis elegans* and the brain of mice (Fang et al., 2019). Spermidine can increase autophagy activity by affecting autophagy-related gene expression and induce mitophagy by inhibiting mTOR and activating adenylate-activated protein kinase (AMPK) (Fan et al., 2017; Madeo et al., 2018). AMPK is an energy sensor in mitochondria, regulated by the cellular AMP/ATP ratio, and is involved in mitophagy in various ways. Deletion of AMPK results in abnormal accumulation of p62 protein and defects in mitophagy in mammals (Yang et al., 2018). In addition, spermidine was reported to induce mitophagy in cultured cell lines of human fibroblasts and cardiomyocytes.

Rapamycin (RAP) and metformin are two FDA-approved mTOR inhibitors with anticancer and antiaging properties. RAP is currently the most important drug for the treatment of mitophagy deficiency (Carosi and Sargeant, 2019). Abnormal levels of mTOR, particularly mTORC1, can lead to excessive mitophagy, and the inhibition of mTOR appears to be a novel approach to promoting mitophagy, possibly improving the symptoms of neurodegenerative diseases (Xu and Brink, 2016). In mutant APP transgenic mouse models, RAP is reported to reduce Aβ pathology, improve cognitive dysfunction, and either slow or block AD progression (Spilman et al., 2010). Metformin stimulates mitophagy by restoring mTOR-dependent autophagy activation and Parkin-mediated mechanisms (Song et al., 2016).

### Mitophagy enhancers in aging, and neurodegenerative diseases

Recent studies have shown that dysfunctional mitochondria are one of the causes of worsening in patients with aging and age-related neurodegenerative diseases. Previous studies have suggested that mitophagy activation may serve as a potential therapeutic chance to remove damaged mitochondria. Mitophagy enhancers remove defective mitochondria and enhance the clearance of mitochondrial debris from affected cells, which could be a therapeutic drug for neurodegenerative diseases. Studies have shown that UA, actinomycin, and tomatine can increase PINK1 and Parkin levels,thereby increasing the clearance of unhealthy mitochondria (Ryu et al., 2016; Fang and Tao, 2020; Esselun et al., 2021). In immortalized primary mouse hippocampal neurons (HT22), treated with mitophagy enhancers such as UA, actinomycin, tomatine, and nicotinamide riboside, the survival rate of HT22 cells, mitochondrial fusion, Mitophagy gene expression was increased, and mitochondrial fragmentation was reduced. Interestingly, among the tested mitophagy enhancers, UA showed the strongest protective effect (Kshirsagar et al., 2021). UA is protective against human Aβ peptide-induced toxicity, and combined treatment of UA with amyloid-β and/or P-tau inhibitors such as green tea extract EGCG has shown a combined therapeutic effect better and better effect, indicating that combination therapy is expected to treat late-onset AD patients (Kshirsagar et al., 2022). UA stimulates mitophagy and improves muscle health in aged animals and clinical models of aging. The study reports the results of a first-in-human clinical trial that after regular oral administration in humans, UA induced improved mitochondrial and cellular Molecular signatures of health (Andreux et al., 2019). To develop potential mitophagy enhancers, further studies are still needed to identify mitophagy enhancers (Pradeepkiran and Reddy, 2020).

Defective mitophagy is a common characteristic of a wide range of neurodegenerative disorders. The autopsy evidence collected from patients with neurodegenerative disorders suggested that dysregulated mitophagy triggers multiple forms of neurodegeneration. These observations can facilitate the search and rationale for screening novel mitophagy-targeted drugs as therapeutic strategies for AD, PD, HD, and ALS. The summary of small molecular compounds that induce mitophagy and their associated mechanisms is given in Table 1.

**Table 1:** Compounds targeting mitophagy and their effects.

**Compounds**	**Mitophagy-related mechanisms**	**Diseases**	**References**
NAD^+^ precursor	Increases the NAD^+^/NADH ratio, promoting mitophagy	AD	Jang et al., 2012; Brown et al., 2014; Scheibye-Knudsen et al., 2014; Fang et al., 2016
Urolithin A	Promotes PINK1-dependent pathway	AD	Ryu et al., 2016; Jayatunga et al., 2021
Actinin	Enhances kinase activity of PINK1 and promotes mitochondrial fission	AD	Burman et al., 2017
Spermidine	Enhances mitophagy	AD/PD/HD/ALS	Eisenberg et al., 2016
Rapamycin	Increases p62 and Parkin translocation to damaged mitochondria	AD/PD/HD/ALS	Spilman et al., 2010; Li et al., 2018
Metformin	Restores Parkin-mediated mitophagy	AD/PD/HD/ALS	Song et al., 2016; Vázquez-Manrique et al., 2016
Deferiprone	Causes iron loss, triggering PINK1/Parkin independent mitophagy	PD	Allen et al., 2013; Devos et al., 2014; Kirienko et al., 2015
Trehalose	Induces mitophagy	AD/PD/HD/ALS	Sarkar et al., 2007

## Outlook

Defective mitochondria cause progressive accumulation of damaged organelles and are characterized as a hallmark of neurodegenerative diseases. This review revealed that mitophagy plays a significant role in maintaining body homeostasis. Healthy mitochondria can provide sufficient energy for neuronal protection and repair mechanisms. Failure of clearing damaged mitochondria can lead to neuronal dysfunction, impairment, and degeneration. Therefore, the elimination of defective mitochondria is essential for the proper functioning of neurons. At present, the existing therapies for neurodegenerative diseases can only relieve the symptoms but cannot cure the disease. Understanding the pathophysiological mechanisms associated with neurodegeneration may help develop pharmacological and nutritional interventions to combat cognitive decline. Although there is no clear candidate therapeutic drug that specifically modulates mitophagy, the development of mitophagy-targeting drugs has increased significantly in the past few years. As the understanding of the mechanisms of mitophagy in different diseases improves, we expect the discovery of new inducing compounds will increase, which will drive further potential clinical studies. Further studies are needed to explore whether specific pharmacological compounds administered early in the disease process can generate meaningful clinical outcomes by targeting multiple processes, and the mechanism of action of these pharmacological compounds will not be restricted to neurons, rather than a single pathway to produce meaningful clinical benefit.

## Author contributions

QW is the main writer of the review and completed the collection and analysis of relevant literature and writing of the first draft of the paper. HX, YY, and SH are involved in the analysis and collation of literature. S-HH and ZZ are the creators and leaders of the project and directed thesis writing. All authors read and agreed with the final text.

## Funding

This research was supported by the National Natural Science Foundation of China (No. 81971088) and the Innovation Project of Shandong Academy of Medical Sciences, Shandong Provincial Natural Science Foundation, China (ZR2019MH090, ZR2018MC008).

## Conflict of interest

The authors declare that the research was conducted in the absence of any commercial or financial relationships that could be construed as a potential conflict of interest.

## Publisher's note

All claims expressed in this article are solely those of the authors and do not necessarily represent those of their affiliated organizations, or those of the publisher, the editors and the reviewers. Any product that may be evaluated in this article, or claim that may be made by its manufacturer, is not guaranteed or endorsed by the publisher.

## References

[B1] Allen G. F. Toth R. James J. Ganley I. G. (2013). Loss of iron triggers PINK1/Parkin-independent mitophagy. EMBO Rep. 14, 1127–1135. 10.1038/embor.2013.168 24176932 PMC3981094

[B2] Amin A. Perera N. D. Beart P. M. Turner B. J. Shabanpoor F. (2020). Amyotrophic lateral sclerosis and autophagy: dysfunction and therapeutic targeting. Cells. 9, 11. 10.3390/cells9112413 33158177 PMC7694295

[B3] Andreux P. A. Blanco-Bose W. Ryu D. Burdet F. Ibberson M. Aebischer P.. (2019). The mitophagy activator urolithin A is safe and induces a molecular signature of improved mitochondrial and cellular health in humans. Nat Metab. 1, 595–603. 10.1038/s42255-019-0073-4 32694802

[B4] Ashrafi G. Schwarz T. L. (2013). The pathways of mitophagy for quality control and clearance of mitochondria. Cell Death Differ. 20, 31–42. 10.1038/cdd.2012.81 22743996 PMC3524633

[B5] Bahn G. Jo D. G. (2019). Therapeutic approaches to alzheimer's disease through modulation of NRF2. Neuromolecular Med. 21, 1–11. 10.1007/s12017-018-08523-5 30617737

[B6] Barreto G. E. Gonzalez J. Capani F. Morales L. (2012). Neuroprotective agents in brain injury: a partial failure? Int. J. Neurosci. 122, 223–226. 10.3109/00207454.2011.648292 22176297

[B7] Bingol B. Sheng M. (2016). Mechanisms of mitophagy: PINK1, Parkin, USP30 and beyond. Free Radical Biol. Med. 100, 210–222. 10.1016/j.freeradbiomed.2016.04.015 27094585

[B8] Brown K.evin D. Maqsood S. Huang J.-Y. Pan Y. Harkcom W. Li W.. (2014). Activation of SIRT3 by the NAD+ precursor nicotinamide riboside protects from noise-induced hearing loss. Cell Metab. 20, 1059–1068. 10.1016/j.cmet.2014.11.003 25470550 PMC4940130

[B9] Burman J. L. Pickles S. Wang C. Sekine S. Vargas J. N. S. Zhang Z.. (2017). Mitochondrial fission facilitates the selective mitophagy of protein aggregates. J. Cell Biol. 216, 3231–3247. 10.1083/jcb.201612106 28893839 PMC5626535

[B10] Cai Q. Tammineni P. (2016). Alterations in mitochondrial quality control in Alzheimer's Disease. Front. Cell Neurosci. 10, 24. 10.3389/fncel.2016.00024 26903809 PMC4746252

[B11] Carosi J. M. Sargeant T. J. (2019). Rapamycin and Alzheimer disease: a double-edged sword? Autophagy. 15, 1460–1462. 10.1080/15548627.2019.1615823 31066320 PMC6613906

[B12] Cen X. Zhang M. Zhou M. Ye L. Xia H. (2021). Mitophagy regulates neurodegenerative diseases. Cells. 10, 8. 10.3390/cells10081876 34440645 PMC8392649

[B13] Chandra A. Sharma A. Calingasan N. Y. White J. M. Shurubor Y. Yang X. W.. (2016). Enhanced mitochondrial biogenesis ameliorates disease phenotype in a full-length mouse model of Huntington's disease. Hum. Mol. Genet. 25, 2269–2282. 10.1093/hmg/ddw095 27008868 PMC5081058

[B14] Chen G. Kroemer G. Kepp O. (2020). Mitophagy: an emerging role in aging and age-associated diseases. Front Cell Dev Biol. 8, 200. 10.3389/fcell.2020.00200 32274386 PMC7113588

[B15] Chiang H. L. Terlecky S. R. Plant C. P. Dice J. F. (1989). A role for a 70-kilodalton heat shock protein in lysosomal degradation of intracellular proteins. Science. 246, 382–385. 10.1126/science.2799391 2799391

[B16] Choo Y. S. Johnson G. V. MacDonald M. Detloff P. J. Lesort M. (2004). Mutant huntingtin directly increases susceptibility of mitochondria to the calcium-induced permeability transition and cytochrome c release. Hum. Mol. Genet. 13, 1407–1420. 10.1093/hmg/ddh162 15163634

[B17] Del Prete D. Suski J. M. Oulès B. Debayle D. Gay A. S. Lacas-Gervais S.. (2017). Localization and processing of the amyloid-β protein precursor in mitochondria-associated membranes. J. Alzheimers. Dis. 55, 1549–1570. 10.3233/JAD-160953 27911326 PMC5181669

[B18] Denisenko T. V. Gogvadze V. Zhivotovsky B. (2021). Mitophagy in carcinogenesis and cancer treatment. Discov Oncol. 12, 58. 10.1007/s12672-021-00454-1 35201480 PMC8777571

[B19] Devos D. Moreau C. Devedjian J. C. Kluza J. Petrault M. Laloux C.. (2014). Targeting chelatable iron as a therapeutic modality in Parkinson's disease. Antioxid. Redox Signal. 21, 195–210. 10.1089/ars.2013.5593 24251381 PMC4060813

[B20] Doxaki C. Palikaras K. (2020). Neuronal mitophagy: friend or foe? Front Cell Dev Biol 8, 611938. 10.3389/fcell.2020.611938 33537304 PMC7848077

[B21] Du F. Yu Q. Yan S. Hu G. Lue L. F. Walker D. G.. (2017). PINK1 signalling rescues amyloid pathology and mitochondrial dysfunction in Alzheimer's disease. Brain. 140, 3233–3251. 10.1093/brain/awx258 29077793 PMC5841141

[B22] Eisenberg T. Abdellatif M. Schroeder S. Primessnig U. Stekovic S. Pendl T.. (2016). Cardioprotection and lifespan extension by the natural polyamine spermidine. Nat. Med. 22, 1428–1438. 10.1038/nm.4222 27841876 PMC5806691

[B23] Esselun C. Theyssen E. Eckert G. P. (2021). Effects of urolithin A on mitochondrial parameters in a cellular model of early Alzheimer disease. Int. J. Mol. Sci. 22, 15. 10.3390/ijms22158333 34361099 PMC8347929

[B24] Evans C. S. Holzbaur E. L. (2020). Degradation of engulfed mitochondria is rate-limiting in Optineurin-mediated mitophagy in neurons. Elife. 9. 10.7554/eLife.50260 31934852 PMC6959996

[B25] Fan J. Yang X. Li J. Shu Z. Dai J. Liu X.. (2017). Spermidine coupled with exercise rescues skeletal muscle atrophy from D-gal-induced aging rats through enhanced autophagy and reduced apoptosis via AMPK-FOXO3a signal pathway. Oncotarget. 8, 17475–17490. 10.18632/oncotarget.15728 28407698 PMC5392263

[B26] Fang E. F. Hou Y. Palikaras K. Adriaanse B. A. Kerr J. S. Yang B.. (2019). Mitophagy inhibits amyloid-β and tau pathology and reverses cognitive deficits in models of Alzheimer's disease. Nat. Neurosci. 22, 401–412. 10.1038/s41593-018-0332-9 30742114 PMC6693625

[B27] Fang E. F. Kassahun H. Croteau D. L. Scheibye-Knudsen M. Marosi K. Lu H.. (2016). NAD+ replenishment improves lifespan and healthspan in ataxia telangiectasia models via mitophagy and DNA repair. Cell Metab. 24, 566–581. 10.1016/j.cmet.2016.09.004 27732836 PMC5777858

[B28] Fang E. F. Lautrup S. Hou Y. Demarest T. G. Croteau D. L. Mattson M. P.. (2017). NAD(+) in aging: molecular mechanisms and translational implications. Trends Mol. Med. 23, 899–916. 10.1016/j.molmed.2017.08.001 28899755 PMC7494058

[B29] Fang E. F. Tao J. (2020). Targeting on the NAD(+)-mitophagy axis to treat cardiovascular disease. Aging Med (Milton). 3, 151–152. 10.1002/agm2.12123 33103034 PMC7574632

[B30] Federico A. Cardaioli E. Pozzo D. a. Formichi P. Gallus P.. (2012). Mitochondria, oxidative stress and neurodegeneration. J. Neurol. Sci. 322, 254–262. 10.1016/j.jns.2012.05.030 22669122

[B31] Feng D. Liu L. Zhu Y. Chen Q. (2013). Molecular signaling toward mitophagy and its physiological significance. Exp. Cell Res. 319, 1697–1705. 10.1016/j.yexcr.2013.03.034 23603281

[B32] Fivenson E. M. Lautrup S. Sun N. Scheibye-Knudsen M. Stevnsner T. Nilsen H.. (2017). Mitophagy in neurodegeneration and aging. Neurochem. Int. 109, 202–209. 10.1016/j.neuint.2017.02.007 28235551 PMC5565781

[B33] Franco-Iborra S. Plaza-Zabala A. Montpeyo M. Sebastian D. Vila M. Martinez-Vicente M.. (2021). (huntingtin) impairs mitophagy in a cellular model of Huntington disease. Autophagy. 17, 672–689. 10.1080/15548627.2020.1728096 32093570 PMC8032238

[B34] Gaetano D. e. Gibellini A. Zanini L. Nasi G. Cossarizza M. Pinti A. M. (2021). Mitophagy and oxidative stress: the role of aging. Antioxidants (Basel). 10, 5. 10.3390/antiox10050794 34067882 PMC8156559

[B35] Gao H. Yang W. Qi Z. Lu L. Duan C. Zhao C.. (2012). DJ-1 protects dopaminergic neurons against rotenone-induced apoptosis by enhancing ERK-dependent mitophagy. J. Mol. Biol. 423, 232–248. 10.1016/j.jmb.2012.06.034 22898350

[B36] Guha S. Fischer S. Johnson G. V. W. Nehrke K. (2020). Tauopathy-associated tau modifications selectively impact neurodegeneration and mitophagy in a novel C. elegans single-copy transgenic model. Mol Neurodegener. 15, 65. 10.1186/s13024-020-00410-7 33168053 PMC7654055

[B37] Gupta V. K. Scheunemann L. Eisenberg T. Mertel S. Bhukel A. Koemans T. S.. (2013). Restoring polyamines protects from age-induced memory impairment in an autophagy-dependent manner. Nat. Neurosci. 16, 1453–1460. 10.1038/nn.3512 23995066

[B38] Hague S. Rogaeva E. Hernandez D. Gulick C. Singleton A. Hanson M.. (2003). A, Early-onset Parkinson's disease caused by a compound heterozygous DJ-1 mutation. Ann. Neurol. 54, 271–274. 10.1002/ana.10663 12891685

[B39] Harding O. Evans C. S. Ye J. Cheung J. Maniatis T. Holzbaur E. L. F.. (2021). ALS- and FTD-associated missense mutations in TBK1 differentially disrupt mitophagy. Proc. Natl. Acad. Sci. USA. 118, 24. 10.1073/pnas.2025053118 34099552 PMC8214690

[B40] Hirota Y. Yamashita S. Kurihara Y. Jin X. Aihara M. Saigusa T.. (2015). Mitophagy is primarily due to alternative autophagy and requires the MAPK1 and MAPK14 signaling pathways. Autophagy. (2015) 11, 332–43. 10.1080/15548627.2015.1023047 25831013 PMC4502654

[B41] Hong X. Liu J. Zhu G. Zhuang Y. Suo H. Wang P.. (2014). Parkin overexpression ameliorates hippocampal long-term potentiation and β-amyloid load in an Alzheimer's disease mouse model. Hum. Mol. Genet. 23, 1056–1072. 10.1093/hmg/ddt501 24105468

[B42] Ingelsson M. Fukumoto H. Newell K. L. Growdon J. H. Hedley–Whyte E. T. Frosch M. P.. (2004). Early Aβ accumulation and progressive synaptic loss, gliosis, and tangle formation in AD brain. Neurology. 62, 925–931. 10.1212/01.WNL.0000115115.98960.37 15037694

[B43] Iorio R. Celenza G. Petricca S. (2021). Mitophagy: molecular mechanisms, new concepts on parkin activation and the emerging role of AMPK/ULK1 axis. Cells. 11. (1). 10.3390/cells11010030 35011593 PMC8750607

[B44] Jang S. Y. Kang H. T. Hwang E. S. (2012). Nicotinamide-induced mitophagy: event mediated by high NAD+/NADH ratio and SIRT1 protein activation. J. Biol. Chem. 287, 19304–19314. 10.1074/jbc.M112.363747 22493485 PMC3365962

[B45] Jayatunga D. P. W. Hone E. Khaira H. Lunelli T. Singh H. Guillemin G. J.. (2021). Therapeutic potential of mitophagy-inducing microflora metabolite, urolithin A for Alzheimer's disease. Nutrients. 13, 11. 10.3390/nu13113744 34836000 PMC8617978

[B46] Jimenez-Sanchez M. Licitra F. Underwood B. R. Rubinsztein D. C. (2017). Huntington's disease: mechanisms of pathogenesis and therapeutic strategies. Cold Spring Harb. Perspect. Med. 7. 10.1101/cshperspect.a024240 27940602 PMC5495055

[B47] Johnson J. A. Johnson D. A. Kraft A. D. Calkins M. J. Jakel R. J. Vargas M. R.. (2008). The Nrf2-ARE pathway: an indicator and modulator of oxidative stress in neurodegeneration. Ann. N. Y. Acad. Sci. 1147, 61–69. 10.1196/annals.1427.036 19076431 PMC2605641

[B48] Kerr J. S. Adriaanse B. A. Greig N. H. Mattson M. P. Cader M. Z. Bohr V. A.. (2017). Mitophagy and Alzheimer's disease: cellular and molecular mechanisms. Trends Neurosci. 40, 151–166. 10.1016/j.tins.2017.01.002 28190529 PMC5341618

[B49] Kirienko N. V. Ausubel F. M. Ruvkun G. (2015). Mitophagy confers resistance to siderophore-mediated killing by Pseudomonas aeruginosa. Proc. Natl. Acad. Sci. USA. 112, 1821–1826. 10.1073/pnas.1424954112 25624506 PMC4330731

[B50] Knott A. B. Perkins G. Schwarzenbacher R. Bossy-Wetzel E. (2008). Mitochondrial fragmentation in neurodegeneration. Nat. Rev. Neurosci. 9, 505–518. 10.1038/nrn2417 18568013 PMC2711514

[B51] Kshirsagar S. Alvir R. V. Pradeepkiran J. A. Hindle A. Vijayan M. Ramasubramaniam B.. (2022). A combination therapy of Urolithin A+EGCG has stronger protective effects than single drug urolithin A in a humanized amyloid beta knockin mice for late-onset alzheimerandrsquos disease. Cells. 11, 2660. 10.3390/cells11172660 36078067 PMC9454743

[B52] Kshirsagar S. Sawant N. Morton H. Reddy A. P. Reddy P. H. (2021). Mitophagy enhancers against phosphorylated Tau-induced mitochondrial and synaptic toxicities in Alzheimer disease. Pharmacol. Res. 174, 105973. 10.1016/j.phrs.2021.105973 34763094 PMC8670983

[B53] Lazarou M. Sliter D. A. Kane L. A. Sarraf S. A. Wang C. Burman J. L.. (2015). The ubiquitin kinase PINK1 recruits autophagy receptors to induce mitophagy. Nature. 524, 309–314. 10.1038/nature14893 26266977 PMC5018156

[B54] Li Q. Gao S. Kang Z. Zhang M. Zhao X. Zhai Y.. (2018). Rapamycin enhances mitophagy and attenuates apoptosis after spinal ischemia-reperfusion injury. Front. Neurosci. 12, 865. 10.3389/fnins.2018.00865 30559639 PMC6286985

[B55] Liu H. Dai C. Fan Y. Guo B. Ren K. Sun T.. (2017). From autophagy to mitophagy: the roles of P62 in neurodegenerative diseases. J. Bioenerg. Biomembr. 49, 413–422. 10.1007/s10863-017-9727-7 28975445

[B56] Liu J. Liu W. Li R. Yang H. (2019). Mitophagy in Parkinson's disease: from pathogenesis to treatment. Cells. 8, 7. 10.3390/cells8070712 31336937 PMC6678174

[B57] Liu L. Feng D. Chen G. Chen M. Zheng Q. Song P.. (2012). Mitochondrial outer-membrane protein FUNDC1 mediates hypoxia-induced mitophagy in mammalian cells. Nat. Cell Biol. 14, 177–185. 10.1038/ncb2422 22267086

[B58] Liu L. Sakakibara K. Chen Q. Okamoto K. (2014). Receptor-mediated mitophagy in yeast and mammalian systems. Cell Res. 24, 787–795. 10.1038/cr.2014.75 24903109 PMC4085769

[B59] Liu X. He J. Chen L. Zhang N. Tang L. Liu X.. (2021). TBK1 variants in Chinese patients with amyotrophic lateral sclerosis. Neurobiol. Aging. 97, 149.e9–149.e15. 10.1016/j.neurobiolaging.2020.07.028 32893041

[B60] Luo Y. Hoffer A. Hoffer B. Mitochondria Q. X. (2015). A therapeutic target for Parkinson's disease? Int. J. Mol. Sci. 16, 20704–20730. 10.3390/ijms160920704 26340618 PMC4613227

[B61] Madeo F. Eisenberg T. Pietrocola F. Kroemer G. (2018). Spermidine in health and disease. Science. 359, 6374. 10.1126/science.aan2788 29371440

[B62] Manczak M. Anekonda T. S. Henson E. Park B. S. Quinn J. Reddy P. H.. (2006). Mitochondria are a direct site of A beta accumulation in Alzheimer's disease neurons: implications for free radical generation and oxidative damage in disease progression. Hum. Mol. Genet. 15, 1437–1449. 10.1093/hmg/ddl066 16551656

[B63] Manczak M. Kandimalla R. Yin X. Reddy P. H. (2018). Hippocampal mutant APP and amyloid beta-induced cognitive decline, dendritic spine loss, defective autophagy, mitophagy and mitochondrial abnormalities in a mouse model of Alzheimer's disease. Hum. Mol. Genet. 27, 1332–1342. 10.1093/hmg/ddy042 29408999 PMC6455948

[B64] Mani S. Swargiary G. Chadha R. (2021). Mitophagy impairment in neurodegenerative diseases: Pathogenesis and therapeutic interventions. Mitochondrion 57, 270–293. 10.1016/j.mito.2021.01.001 33476770

[B65] Manocha G. D. Floden A. M. Rausch K. Kulas J. A. McGregor B. A. Rojanathammanee L.. (2016). APP regulates microglial phenotype in a mouse model of Alzheimer's disease. J. Neurosci. 36, 8471–8486. 10.1523/JNEUROSCI.4654-15.2016 27511018 PMC4978805

[B66] Marinkovi,ć M. Novak I. (2021). A brief overview of BNIP3L/NIX receptor-mediated mitophagy. FEBS Open Bio. 11, 3230–3236. 10.1002/2211-5463.13307 34597467 PMC8634856

[B67] Mattson M. P. Gleichmann M. Cheng A. (2008). Mitochondria in neuroplasticity and neurological disorders. Neuron. 60, 748–766. 10.1016/j.neuron.2008.10.010 19081372 PMC2692277

[B68] Millecamps S. Julien J. P. (2013). Axonal transport deficits and neurodegenerative diseases. Nat. Rev. Neurosci. 14, 161–176. 10.1038/nrn3380 23361386

[B69] Minowa-Nozawa A. Nozawa T. Okamoto-Furuta K. Kohda H. Nakagawa I. (2017). Rab35 GTPase recruits NDP52 to autophagy targets. EMBO J. 36, 2790–2807. 10.15252/embj.201796463 28848034 PMC5599792

[B70] Mizushima N. Komatsu M. (2011). Autophagy: renovation of cells and tissues. Cell. 147, 728–741. 10.1016/j.cell.2011.10.026 22078875

[B71] Narendra D. Kane L. A. Hauser D. N. Fearnley I. M. Youle R. J. (2010). p62/SQSTM1 is required for Parkin-induced mitochondrial clustering but not mitophagy; VDAC1 is dispensable for both. Autophagy. 6, 1090–1106. 10.4161/auto.6.8.13426 20890124 PMC3359490

[B72] Nguyen T. N. Padman B. S. Lazarou M. (2016). Deciphering the molecular signals of PINK1/parkin mitophagy. Trends Cell Biol. 26, 733–744. 10.1016/j.tcb.2016.05.008 27291334

[B73] Novak I. Kirkin V. McEwan D. G. Zhang J. Wild P. Rozenknop A.. (2010). Nix is a selective autophagy receptor for mitochondrial clearance. EMBO Rep. 11, 45–51. 10.1038/embor.2009.256 20010802 PMC2816619

[B74] Oliver D. M. A. Reddy P. H. (2019). Small molecules as therapeutic drugs for Alzheimer's disease. Mol. Cell. Neurosci. 96, 47–62. 10.1016/j.mcn.2019.03.001 30877034 PMC6510253

[B75] Onishi M. Yamano K. Sato M. Matsuda N. Okamoto K. (2021). Molecular mechanisms and physiological functions of mitophagy. EMBO J. 40, e104705. 10.15252/embj.2020104705 33438778 PMC7849173

[B76] Palikaras K. Lionaki E. Tavernarakis N. (2018). Mechanisms of mitophagy in cellular homeostasis, physiology and pathology. Nat. Cell Biol. 20, 1013–1022. 10.1038/s41556-018-0176-2 30154567

[B77] Palikaras K. Tavernarakis N. (2012). Mitophagy in neurodegeneration and aging. Front. Genet. 3, 297. 10.3389/fgene.2012.00297 23267366 PMC3525948

[B78] Pellerin L. Bouzier-Sore A. K. Aubert A. Serres S. Merle M. Costalat R.. (2007). Activity-dependent regulation of energy metabolism by astrocytes: an update. Glia. 55, 1251–1262. 10.1002/glia.20528 17659524

[B79] Pickles S. Vigié P. Youle R. J. (2018). Mitophagy and quality control mechanisms in mitochondrial maintenance. Curr. Biol. 28, R170–r185. 10.1016/j.cub.2018.01.004 29462587 PMC7255410

[B80] Podvin S. Reardon H. T. Yin K. Mosier C. Hook V. (2019). Multiple clinical features of Huntington's disease correlate with mutant HTT gene CAG repeat lengths and neurodegeneration. J. Neurol. 266, 551–564. 10.1007/s00415-018-8940-6 29956026

[B81] Pradeepkiran J. A. Reddy P. H. (2020). Defective mitophagy in Alzheimer's disease. Ageing Res. Rev. 64, 101191. 10.1016/j.arr.2020.101191 33022416 PMC7710581

[B82] Puri R. Suzuki T. Yamakawa K. Ganesh S. (2009). Hyperphosphorylation and aggregation of Tau in laforin-deficient mice, an animal model for Lafora disease. J. Biol. Chem. 284, 22657–22663. 10.1074/jbc.M109.009688 19542233 PMC2755673

[B83] Raghunath A. Sundarraj K. Nagarajan R. Arfuso F. Bian J. Kumar A. P.. (2018). Antioxidant response elements: Discovery, classes, regulation and potential applications. Redox Biol. 17, 297–314. 10.1016/j.redox.2018.05.002 29775961 PMC6007815

[B84] Rosen D. R. Siddique T. Patterson D. Figlewicz D. A. Sapp P. Hentati A.. (1993). Mutations in Cu/Zn superoxide dismutase gene are associated with familial amyotrophic lateral sclerosis. Nature. 362, 59–62. 10.1038/362059a0 8446170

[B85] Ross C. A. Tabrizi S. J. (2011). Huntington's disease: from molecular pathogenesis to clinical treatment. Lancet Neurol. 10, 83–98. 10.1016/S1474-4422(10)70245-3 21163446

[B86] Rossmann M. P. Dubois S. M. Agarwal S. Zon L. I. (2021). Mitochondrial function in development and disease. Dis. Model. Mech. 14, 6. 10.1242/dmm.048912 34114603 PMC8214736

[B87] Rowland L. P. Shneider N. A. (2001). Amyotrophic lateral sclerosis. N. Engl. J. Med. 344, 1688–1700. 10.1056/NEJM200105313442207 11386269

[B88] Ryan B. J. Hoek S. Fon E. A. Wade-Martins R. (2015). Mitochondrial dysfunction and mitophagy in Parkinson's: from familial to sporadic disease. Trends Biochem. Sci. 40, 200–210. 10.1016/j.tibs.2015.02.003 25757399

[B89] Ryu D. Mouchiroud L. Andreux P. A. Katsyuba E. Moullan N. Nicolet-Dit-Félix A. A.. (2016). induces mitophagy and prolongs lifespan in elegans C, and increases muscle function in rodents. Nat. Med. 22, 879–888. 10.1038/nm.4132 27400265

[B90] Sandoval H. Yao C. K. Chen K. Jaiswal M. Donti T. Lin Y. Q.. (2014). Mitochondrial fusion but not fission regulates larval growth and synaptic development through steroid hormone production. Elife. 3. 10.7554/eLife.03558.023 25313867 PMC4215535

[B91] Sarkar S. Davies J. E. Huang Z. Tunnacliffe A. Rubinsztein D. C. (2007). Trehalose, a novel mTOR-independent autophagy enhancer, accelerates the clearance of mutant huntingtin and alpha-synuclein. J. Biol. Chem. 282, 5641–5652. 10.1074/jbc.M609532200 17182613

[B92] Scheibye-Knudsen M. Sarah J. Fang Evandro F. Iyama T. Ward T. Wang J.. (2014). A high-fat diet and NAD+ activate Sirt1 to rescue premature aging in cockayne syndrome. Cell Metab. 20, 840–855. 10.1016/j.cmet.2014.10.005 25440059 PMC4261735

[B93] Schönfeld P. Reiser G. (2013). Why does brain metabolism not favor burning of fatty acids to provide energy? - reflections on disadvantages of the use of free fatty acids as fuel for brain. J. Cerebral Blood Flow Metabol. 33, 1493–1499. 10.1038/jcbfm.2013.128 23921897 PMC3790936

[B94] Smith E. F. Shaw P. J. Vos D. (2019). The role of mitochondria in amyotrophic lateral sclerosis. Neurosci. Lett. 710, 132933. 10.1016/j.neulet.2017.06.052 28669745

[B95] Song Y. M. Lee W. K. Lee Y. H. Kang E. S. Cha B. S. Lee B. W.. (2016). Metformin restores parkin-mediated mitophagy, suppressed by Cytosolic p53. Int. J. Mol. Sci. 17. 10.3390/ijms17010122 26784190 PMC4730363

[B96] Spilman P. Podlutskaya N. Hart M. J. Debnath J. Gorostiza O. Bredesen D.. (2010). Inhibition of mTOR by rapamycin abolishes cognitive deficits and reduces amyloid-beta levels in a mouse model of Alzheimer's disease. PLoS ONE. 5, e9979. 10.1371/journal.pone.0009979 20376313 PMC2848616

[B97] Stavoe A. K. H. Holzbaur E. L. F. (2019). Autophagy in neurons. Annu. Rev. Cell Dev. Biol. 35, 477–500. 10.1146/annurev-cellbio-100818-125242 31340124 PMC6996145

[B98] Strappazzon F. Nazio F. Corrado M. Cianfanelli V. Romagnoli A. Fimia G. M.. (2015). AMBRA1 is able to induce mitophagy via LC3 binding, regardless of PARKIN and p62/SQSTM1. Cell Death Differ. 22, 419–432. 10.1038/cdd.2014.139 25215947 PMC4326570

[B99] Sukhorukov V. Voronkov D. Baranich T. Mudzhiri N. Magnaeva A. Illarioshkin S.. (2021). Impaired mitophagy in neurons and glial cells during aging and age-related disorders. Int. J. Mol. Sci. 22. (19). 10.3390/ijms221910251 34638589 PMC8508639

[B100] Swerdlow N. S. Wilkins H. M. (2020). Mitophagy and the Brain. Int. J. Mol. Sci. 21. (24). 10.3390/ijms21249661 33352896 PMC7765816

[B101] Tak Y. J. Park J. H. Rhim H. Kang S. (2020). ALS-related mutant SOD1 aggregates interfere with mitophagy by sequestering the autophagy receptor optineurin. Int. J. Mol. Sci. 21. (20). 10.3390/ijms21207525 33065963 PMC7590160

[B102] Valente E. M. Abou-Sleiman P. M. Caputo V. Muqit M. M. Harvey K. Gispert S.. (2004). Hereditary early-onset Parkinson's disease caused by mutations in PINK1. Science. 304, 1158–1160. 10.1126/science.1096284 15087508

[B103] Vázquez-Manrique R. P. Farina F. Cambon K. Dolores Sequedo M. Parker A. J. Millán J. M.. (2016). AMPK activation protects from neuronal dysfunction and vulnerability across nematode, cellular and mouse models of Huntington's disease. Hum. Mol. Genet. 25, 1043–1058. 10.1093/hmg/ddv513 26681807 PMC4764188

[B104] Wang W. Zhao F. Ma X. Perry G. Zhu X. (2020). Mitochondria dysfunction in the pathogenesis of Alzheimer's disease: recent advances. Mol. Neurodegener. 15, 30. 10.1186/s13024-020-00376-6 32471464 PMC7257174

[B105] Wang X. Becker K. Levine N. Zhang M. Lieberman A. P. Moore D. J.. (2019). Pathogenic alpha-synuclein aggregates preferentially bind to mitochondria and affect cellular respiration. Acta Neuropathol Commun. 7, 41. 10.1186/s40478-019-0696-4 30871620 PMC6419482

[B106] Wong Y. C. Holzbaur E. L. (2014). The regulation of autophagosome dynamics by huntingtin and HAP1 is disrupted by expression of mutant huntingtin, leading to defective cargo degradation. J. Neurosci. 34, 1293–1305. 10.1523/JNEUROSCI.1870-13.2014 24453320 PMC3898289

[B107] Wong Y. C. Holzbaur E. L. (2015). Autophagosome dynamics in neurodegeneration at a glance. J. Cell Sci. 128, 1259–1267. 10.1242/jcs.161216 25829512 PMC4379723

[B108] Xu L. Brink M. (2016). mTOR, cardiomyocytes and inflammation in cardiac hypertrophy. Biochimica et Biophysica Acta (BBA) - Molecular Cell Research. 7, 1894–1903. 10.1016/j.bbamcr.2016.01.003 26775585

[B109] Yamano K. Youle R. J. (2013). PINK1 is degraded through the N-end rule pathway. Autophagy. 9, 1758–1769. 10.4161/auto.24633 24121706 PMC4028335

[B110] Yang P. Ling L. Sun W. Yang J. Zhang L. Chang G.. (2018). Ginsenoside Rg1 inhibits apoptosis by increasing autophagy via the AMPK/mTOR signaling in serum deprivation macrophages. Acta Biochim Biophys Sin (Shanghai). 50, 144–155. 10.1093/abbs/gmx136 29324976

[B111] Youle R. J. van der Bliek A. M. (2012). Mitochondrial fission, fusion, and stress. Science. 337, 1062–1065. 10.1126/science.1219855 22936770 PMC4762028

[B112] Zhang F. Ström A. L. Fukada K. Lee S. Hayward L. J. Zhu H.. (2007). Interaction between familial amyotrophic lateral sclerosis (ALS)-linked SOD1 mutants and the dynein complex. J. Biol. Chem. 282, 16691–16699. 10.1074/jbc.M609743200 17403682

[B113] Zhang J. Ney P. A. induces N. I. (2008). mitochondrial autophagy in reticulocytes. Autophagy. 4, 354–356. 10.4161/auto.5552 18623629

[B114] Zhou M. Ottenberg G. Sferrazza G. F. Hubbs C. Fallahi M. Rumbaugh G.. (2015). Neuronal death induced by misfolded prion protein is due to NAD+ depletion and can be relieved in vitro and in vivo by NAD+ replenishment. Brain. 138, 992–1008. 10.1093/brain/awv002 25678560 PMC4840455

[B115] Zhu X. Perry G. Smith M. A. Wang X. (2013). Abnormal mitochondrial dynamics in the pathogenesis of Alzheimer's disease. J. Alzheimers Dis. 33, S253–62. 10.3233/JAD-2012-129005 22531428 PMC4097015

